# Tumor–lymph cross-plane projection reveals spatial relationship features: a ResNet-CBAM model for prognostic prediction in esophageal cancer

**DOI:** 10.3389/fonc.2025.1567238

**Published:** 2025-03-21

**Authors:** Jiayang Xu, Chen Huang, Qianshun Chen, Jieyang Wang, Yuyu Lin, Wei Tang, Wei Shen, Xunyu Xu

**Affiliations:** ^1^ Shengli Clinical Medical College of Fujian Medical University, Fuzhou, Fujian, China; ^2^ Thoracic Surgery Department of Fujian Provincial Hospital, Fuzhou, Fujian, China; ^3^ Thoracic Surgery Department of Fuzhou University Affiliated Provincial Hospital, Fuzhou, Fujian, China

**Keywords:** esophageal cancer, prognostic prediction, deep learning, ResNet, CBAM, cross-plane projection

## Abstract

**Background:**

Prognostic models for esophageal cancer based on contrast-enhanced chest CT can aid thoracic surgeons in developing personalized treatment plans to optimize patient outcomes. However, the extensive lymphatic drainage and early lymph node metastasis of the esophagus present significant challenges in extracting and analyzing meaningful lymph node characteristics. Previous studies have primarily focused on tumor and lymph node features separately, overlooking spatial correlations such as position, direction, and volumetric ratio.

**Methods:**

A total of 285 patients who underwent radical resection surgery at Fujian Provincial Hospital from 2018 to 2022 were retrospectively analyzed. This study introduced a tumor–lymph node projection plane, created by projecting lymph node ROIs onto the tumor ROI plane. A ResNet-CBAM model, integrating a residual convolutional neural network with a CBAM attention module, was employed for feature extraction and survival prediction. The PJ group utilized tumor–lymph node projection planes as training data, while the TM and ZC groups utilized tumor ROIs and concatenated images of tumor and lymph node ROIs, respectively, as controls. Additional comparisons were made with traditional machine learning models (support vector machines, logistic regression, and K-nearest neighbors). Survival outcomes (median, 1-year, 3-year, 5-year) were used as target labels to evaluate model performance in distinguishing high-risk patients and predicting both short- and long-term survival.

**Results:**

In the PJ group, the ResNet-CBAM model achieved accuracy rates of 0.766, 0.981, 0.883, and 0.778 for predicting median, 1-year, 3-year, and 5-year survival, respectively. Its corresponding AUC values for 1-, 3-, and 5-year survival were 0.992, 0.913, and 0.835. Kaplan–Meier survival analysis revealed significant differences between high- and low-risk groups identified by the model. The ResNet-CBAM model outperformed those in the TM and ZC groups in distinguishing high-risk patients and predicting both short- and long-term survival. Compared to machine learning models, it demonstrated superior performance in long-term survival prediction.

**Conclusion:**

The ResNet-CBAM model trained on tumor–lymph projection planes effectively distinguished high-risk esophageal cancer patients and outperformed traditional models in predicting survival outcomes. By capturing spatial relationships between tumors and lymph nodes, it demonstrated enhanced predictive efficiency.

## 1 Introduction

Esophageal cancer, as a complex gastrointestinal tumor with a poor prognosis, has become a major global disease burden (1). Patients with esophageal cancer often require complex, multimodal treatment regimens, including surgery, chemotherapy, and radiotherapy. Therefore, tailoring individualized treatment plans based on the specific condition of each patient is essential for maximizing clinical outcomes and improving prognosis. Machine learning and deep learning models have been shown to improve doctors’ ability to predict the prognosis of esophageal cancer. Simpler models rely on clinical history data from public databases to predict the clinical stage and survival prognosis of esophageal cancer (2, 3). Convolutional neural networks enable models to extract image features from esophageal cancer CT and digital pathology, which have been shown to better predict the risk of survival (4–7). As research has advanced, studies have increasingly focused on the critical role of lymph node characteristics in esophageal cancer. For surgical treatment and radiation therapy, deep learning models have demonstrated the ability to accurately identify and segment malignant lymph nodes (8–10) or predict lymph node metastasis based on tumor images (11–13). While deep learning and machine learning have performed exceptionally well in these tasks, most approaches still extract tumor and lymph node images separately and included them in models as independent features. This method disrupts the spatial relationships between tumors and lymph nodes, potentially limiting prognostic accuracy (14).

Esophageal cancer is characterized by early lymph node metastasis, and the imaging features of lymph nodes in the esophageal drainage area are crucial for assessing survival risk and predicting prognosis. However, esophageal lymphatic drainage is extensive and complex, with frequent occurrences of skip metastases and contralateral metastases. Suspicious metastatic lymph nodes on CT images are often distributed across multiple sectional planes. Effectively extracting and analyzing lymph node features in esophageal cancer is crucial for advancing CT imaging-based prognostic predictions; however, it remains a significant challenge.

The imaging characteristics of tumors and lymph nodes can be categorized into local and global features. Local features, such as image texture and boundary morphology, are closely associated with the pathological classification and histological characteristics of tumors (15). To analyze these features effectively, Convolutional Neural Networks (CNNs) have been widely applied in medical image analysis, including the diagnosis and prognosis of esophageal cancer. CNNs can effectively extract local features and high-dimensional representations from computerized tomography (CT) images. However, they often overlook global features of tumors and lymph nodes, such as volume proportions, relative positions, and morphologies. These global features may offer insights into tumor invasiveness, metastatic pathways, and other behavioral characteristics. Therefore, developing a method to transform spatial relationship features into more easily extractable planar features is essential.

Interestingly, in fields such as computer vision, effectively modeling and preserving the spatial relationships of regions of interest (ROI) extracted from different planes is a critical task. Projection-based techniques provide robust methods for integrating spatial features across multiple planes, enabling more comprehensive analysis and feature representations. In this study, the original CT images were transformed into the tumor–lymph projecting plane. This process involved extracting tumor and lymph node ROIs from different CT scan slices and projecting the lymph node ROIs onto the tumor ROI slice along the vertical axis. The projection plane combined tumor and lymph node images while preserving their spatial relationships in the sagittal and coronal axes. A deep learning model of residual CNN Residual Network (ResNet) with the self-attention module Convolutional Block Attention Module (CBAM) was trained to predict postoperative survival based on follow-up data. ResNet50 hierarchically extracted local features through convolution operations, while CBAM efficiently aggregated spatial and global features. Since the spatial relationships between tumors and lymph nodes are associated with postoperative prognosis, the ResNet-CBAM model was well-suited for capturing these features.

## 2 Materials and methods

### 2.1 Data source

Data from 300 patients who underwent radical resection surgery (endoscopic esophageal cancer segment + upper digestive tract reconstruction + operative lymph node dissection) were retrospectively collected at Fujian Provincial Hospital from 2018 to 2022. All procedures complied with the ethical standards of the ethics committee on human experimentation at Fujian Provincial Hospital (K2022-09-025).

### 2.2 Exclusion criteria

The exclusion criteria include the following:

1. Pathological types of nonesophageal squamous cell carcinoma or esophageal adenocarcinoma, as confirmed by histopathology;
2. Receiving preoperative radiotherapy or combined preoperative radiotherapy and systemic chemotherapy;
3. Multiple primary esophageal cancers, upper cervical esophageal cancer, suspected distant metastasis, or complicated with other tumors;
4. Artifacts or noise in the enhanced image that remain difficult to eliminate after filtering; and
5. Missing clinical, pathological, or follow-up data.

### 2.3 Sample statistics

Three patients had poor-quality CT images (with noticeable noise persisting after median filtering or noise cancellation optimization). In addition, eight patients and their families could not be contacted, and four deceased patients had uncertain survival times due to inconsistent reports from family members during the follow-up. Ultimately, a total of 285 patients were included in this study (**Table 1**).

**Table 1:** Clinical and pathological data of the included samples.

	(category)	N=285 [mean ± std or n (%)]
**Age (year)**		61.00 ± 8.90
**Gender**	Male	209 (73.33%)
	Female	76 (26.67%)
**Tumor Location**	Upper esophagus	42 (14.74%)
	Middle esophagus	169 (59.30%)
	Lower esophagus	74 (25.96%)
**Pathology Grade**	Grade I	20 (7.02%)
	Grade II	221 (77.54%)
	Grade III	44 (15.44%)
**Vascular Invasion**	Occur	179 (62.81%)
	None	106 (37.19%)
**T stage**	T1	4 (1.40%)
	T2	58 (20.35%)
	T3	218 (76.50)
	T4	5 (1.75%)
**N stage**	N0	130 (45.62%)
	N1	92 (32.28%)
	N2	50 (17.54%)
	N3	13 (4.56%)
**TNM stage**	Ib	3 (1.05%)
	IIa	64 (22.46%)
	IIb	64 (22.46%)
	IIIa	13 (4.56%)
	IIIb	125 (43.86%)
	IVa	16 (5.61%)
**Radiotherapy**	Occur	225 (78.95%)
	None	60 (21.05%)
**Chemotherapy**	Occur	122 (42.81%)
	None	163 (57.19%)
**Dead event**	Dead	154 (54.04%)
	Survival	131 (46.96%)
**Survival time/** **Follow-up time (month)**		53.00 ± 30.84

### 2.4 Image acquisition and preprocessing procedure

A GE Lightspeed 64-slice spiral CT was used to obtain a 512-pixel × 512-pixel matrix for every 3 mm scan. The scanning range extended from the thoracic entrance to the lower edge of both kidneys. Contrast-enhanced scanning was performed by injecting a contrast agent (1–1.5 ml/kg, iopromide injection) with a high-pressure syringe at a flow rate of 3.0 ml/s.

Image segmentation was performed using 3D Slicer software (version 5.0.2) on Digital Imaging and Communications in Medicine (DICOM) format files of primary CT images. A median image filter was applied for denoising, and ROIs were delineated on images with inappropriate windows, referencing enhanced scanning. Threshold procedures were applied to remove pixels with CT values below − 50 HU and above + 300 HU, minimizing interference from surrounding tissues and esophageal contents. Areas where the esophageal wall exceeded 5 mm were considered tumor ROIs. Megascopically enlarged lymph node groups were delineated as lymph ROIs, excluding vascular connective tissue with similar morphology. The preprocessing procedures were independently performed by a senior thoracic surgeon (with more than 10 years of experience in diagnosing esophageal cancer) with the help of automatic delineation tools. Contoured images were then reviewed by another senior surgeon under a blinded assessment.

Data reading and model building were conducted in a Python-based PyTorch environment. The pynrrd package was used to read DICOM files of CT images, extracting pixel grayscale matrices and ROI masks. All grayscale matrices were resampled to 224-pixel × 224-pixel matrix for every 3 mm scan. Tabulated medical record data were imported using the NumPy package.

### 2.5 Tumor–lymph projecting plane

**Figure 1:**
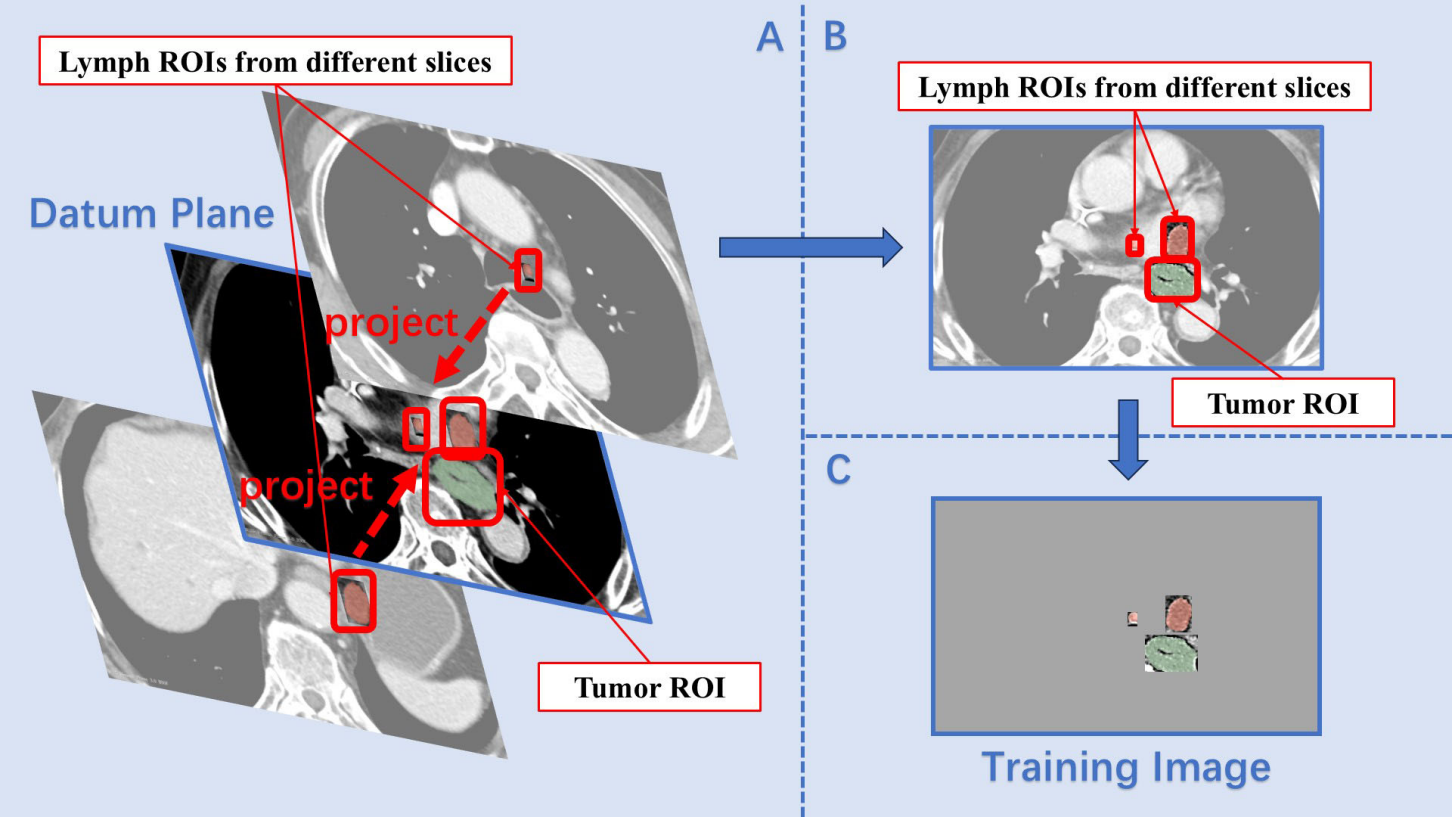
Construction of the projection plane. **(A)** The slice containing the max size tumor ROI was set as the datum plane, and lymph ROIs from different slices were sequentially projected onto it. **(B)** On the datum plane, the relative size and position features between the tumor ROI and lymph ROIs were preserved. **(C)** After masking, the final training image contained only the ROIs.

The tumor–lymph projection plane extracted all lymph node ROIs from each patient’s CT image and projected them onto a specific slice containing the tumor ROI. Since the initial delineation using 3D Slicer had already differentiated between tumor and lymph node ROIs, the main challenge in the projection process was resolving instances where the same lymph node appeared in different scan planes. To address this, the breadth-first search algorithm was employed to identify all ROIs within a complete lymph node group (16). These ROIs were then assigned a new value different from the original marker before being masked. This process was repeated several times until all lymph node ROIs were successfully overwritten. In certain series of CT images, lymph node groups were sorted according to the total amount of pixels of the ROIs in each group. Subsequently, the max-sized ROI from each group was extracted and sequentially placed in a list. The slice containing the max-sized tumor ROI was designated as the datum plane. The projection prodecure involved creating a mask for the blank areas of the datum plane, cropping the lymph node ROIs according to this mask, and then adding the processed lymph ROIs onto the datum plane (**Figure 1**). As all lymph ROIs in the list were projected onto the datum plane in sequence, ROIs from different CT slices were combined into a single two-dimensional image, preserving the majority of image features and spatial information. The images processed using this method formed the projecting group (PJ group).

### 2.6 Image preprocessing in control groups

**Figure 2:**
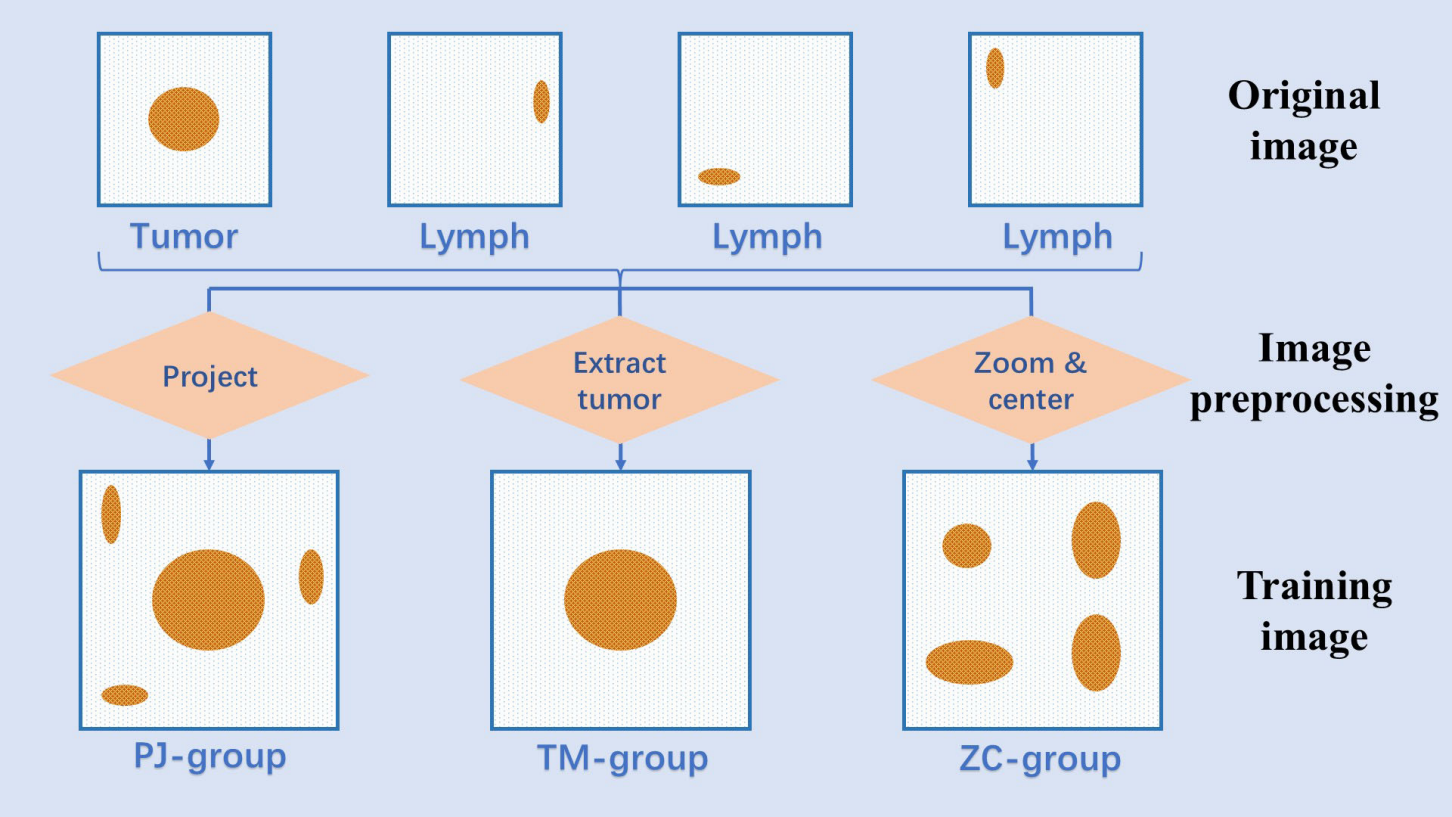
Image preprocessing of all groups.

The sets containing only tumor ROIs were designated as training data for the tumor group (TM group). Meanwhile, to evaluate the impact of projection planes on model performance, a control set containing only local features was required. Using the breadth-first search algorithm, all lymph node ROIs were identified as described above. Lymph node ROIs and tumor ROIs from a series of CT images were then cropped to the center of each slice and resized to a uniform scale. These ROIs were sequentially stitched together onto the same image, effectively eliminating differences in spatial features within the zooming and centering group (ZC group) (**Figure 2**).

### 2.7 Dataset and ResNet-CBAM model

The training images for the PJ, TM, and ZC groups were created following the aforementioned image preprocessing procedures. The model training tasks in this study primarily involved predicting survival risk and survival prognosis. For the task of predicting patient survival risk, samples were categorized into high- and low-risk groups based on the median survival time of the total sample. Specifically, samples with a survival time exceeding the median survival time were labeled as “1”, while the remaining samples were labeled as “0”. For the survival prognosis prediction task, samples were classified according to survival durations of 1, 3, and 5 years and were similarly labeled as “1” and “0”. Image enhancement was applied using random flipping (50% probability) and random rotation (− 60° to +60°). Following this procedure, the number of positive samples was doubled. Negative samples were scaled accordingly to maintain a balanced composition between positive and negative samples. Based on the universal patient coding used in both CT notes and medical records, each patient’s sample image was set as the input, while the corresponding label in tensor form was assigned as the output label. The total dataset was randomly split into a training set and a testing set at a 7:3 ratio using the cross-validation method.

**Figure 3:**
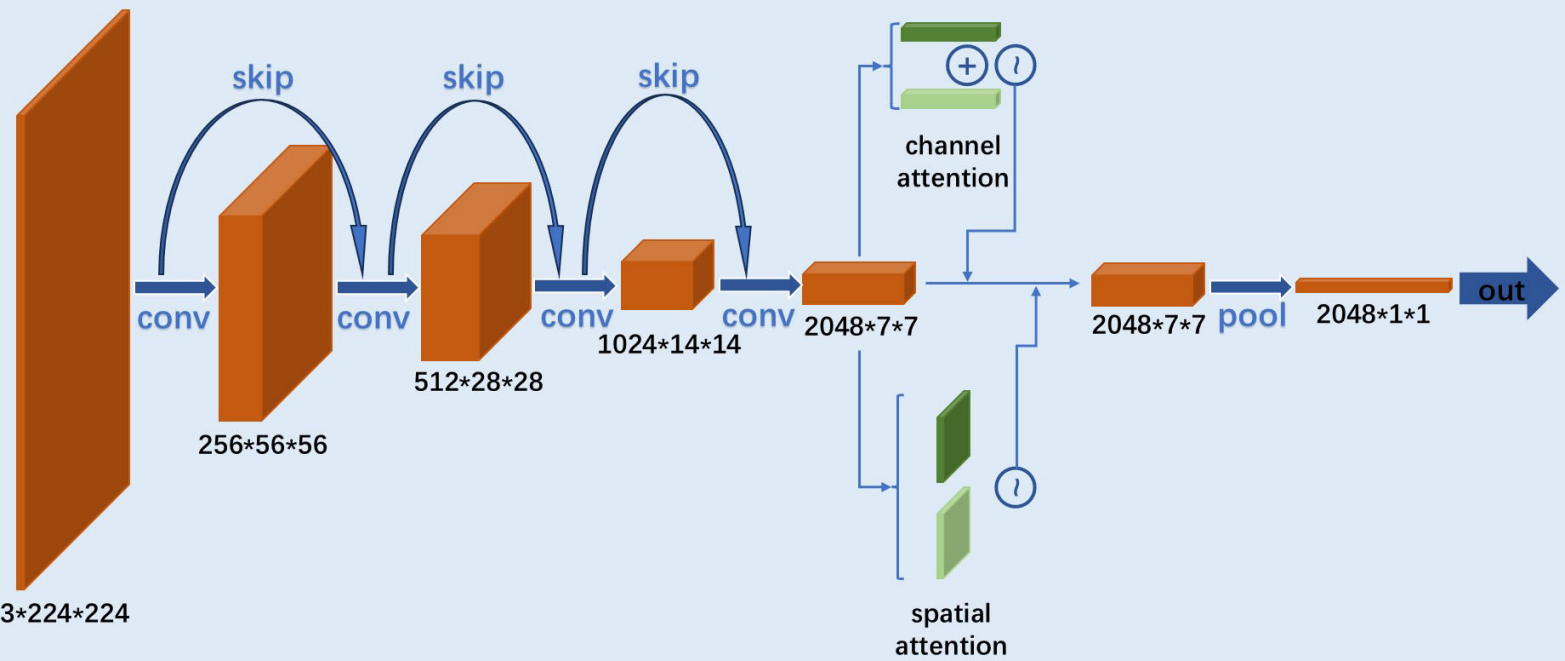
Structural diagram of ResNet-CBAM model.

The main model was a 50-layer ResNet CNN with an incorporated CBAM module (**Figure 3**). ResNet50 is a commonly used convolutional neural network that optimizes gradient descent through a residual mechanism (17). Meanwhile, CBAM serves as an enhancement module to improve the model’s attention to both channel and spatial information (18), addressing the limited ability of CNN to extract global and spatial features effectively. For the feature maps extracted by the CNN, CBAM applied two consecutive weight enhancement operations. First, global average pooling and global maximum pooling are used to extract features along the channel dimension. The two parts of channel features were fused into channel weights through a fully connected layer. In the second step, feature maps underwent max pooling and mean pooling similarly but were then processed by a convolutional layer to obtain spatial weights. Feature maps sequentially receiving channel and spatial weight adjustments were then passed through a fully connected network with nonlinear activation. The cross-entropy function was used to compute the loss value, with L2 regularization applied at a coefficient of 1*e*−4. The Adaptive Moment Estimation (ADAM) optimizer was employed for parameter updates, with learning rate set at 5*e*−5. Given the complexity and robustness of the concatenated model, ridge regularization was used to mitigate overfitting. Considering the small sample size, a smaller batch sizes was selected (for batch size = 4). The model underwent 50 training epochs, and results were recorded.

### 2.8 Preprocessing and training on a mechanical model

In further studies, the training model used by the PJ group was compared horizontally with other machine learning models. The comparison models adopted labels calibrated using the same methods described above as fitting targets, with ResNet50 trained as the feature extractor. Imaging feature scores were extracted using the trained ResNet50 network for tumor ROIs and the three largest lymph node ROIs. For samples in which no suspected positive lymph nodes were identified in the CT images, blank images were used to supplement the training images, and feature scores were similarly obtained. These four imaging scores for each sample formed a feature vector, which served as the independent variable for the machine learning models, while the target labels remained consistent with the objectives of ResNet50. The machine learning models used as controls included support vector machines (SVM), logistic regression (LR), and K-nearest neighbors (KNN), all imported from the scikit-learn library.

### 2.9 Model evaluation

Scores (including accuracy, precision, specificity, recall, and F1-score) evaluating the outputs on the testing set were calculated using the confusion matrix. For the task of predicting median survival, samples were divided according to the prediction results of the model, and the Kaplan–Meier (K-M) survival curve was generated using RStudio based on the original survival events and survival time of the samples. The log-rank test was used to assess whether there was a statistically significant difference in survival risk between the high- and low-risk groups classified by the model. For the task of predicting survival time (1, 3, and 5 years), the ROC curve, plotted using RStudio, provided a more visual presentation.

## 3 Results

### 3.1 ResNet-CBAM model trained on PJ, TM, and ZC group datasets for predicting median survival

From **Table 2**, it is evident that the accuracy of the model trained on the PJ group dataset was significantly higher than that of models trained on the TM and ZC group datasets (**Table 2**). This suggests that the PJ group model more accurately differentiated between patients with low survival risk (survival time exceeding the median survival time) and high survival risk (survival time below the median survival time). Meanwhile, considering the performance of each group in terms of precision, specificity, recall, and F1-score, the PJ group model also demonstrated superior sensitivity and specificity compared to other models.

**Table 2:** Performance of ResNet-CBAM in predicting median survival in the PJ, TM, and ZC groups.

	PJ group	TM group	ZC group
**Accuracy**	0.849	0.765	0.635
**Precision**	0.847	0.822	0.670
**Specificity**	0.845	0.852	0.732
**Recall**	0.853	0.678	0.538
***F*1-score**	0.850	0.743	0.597

**Figure 4:**
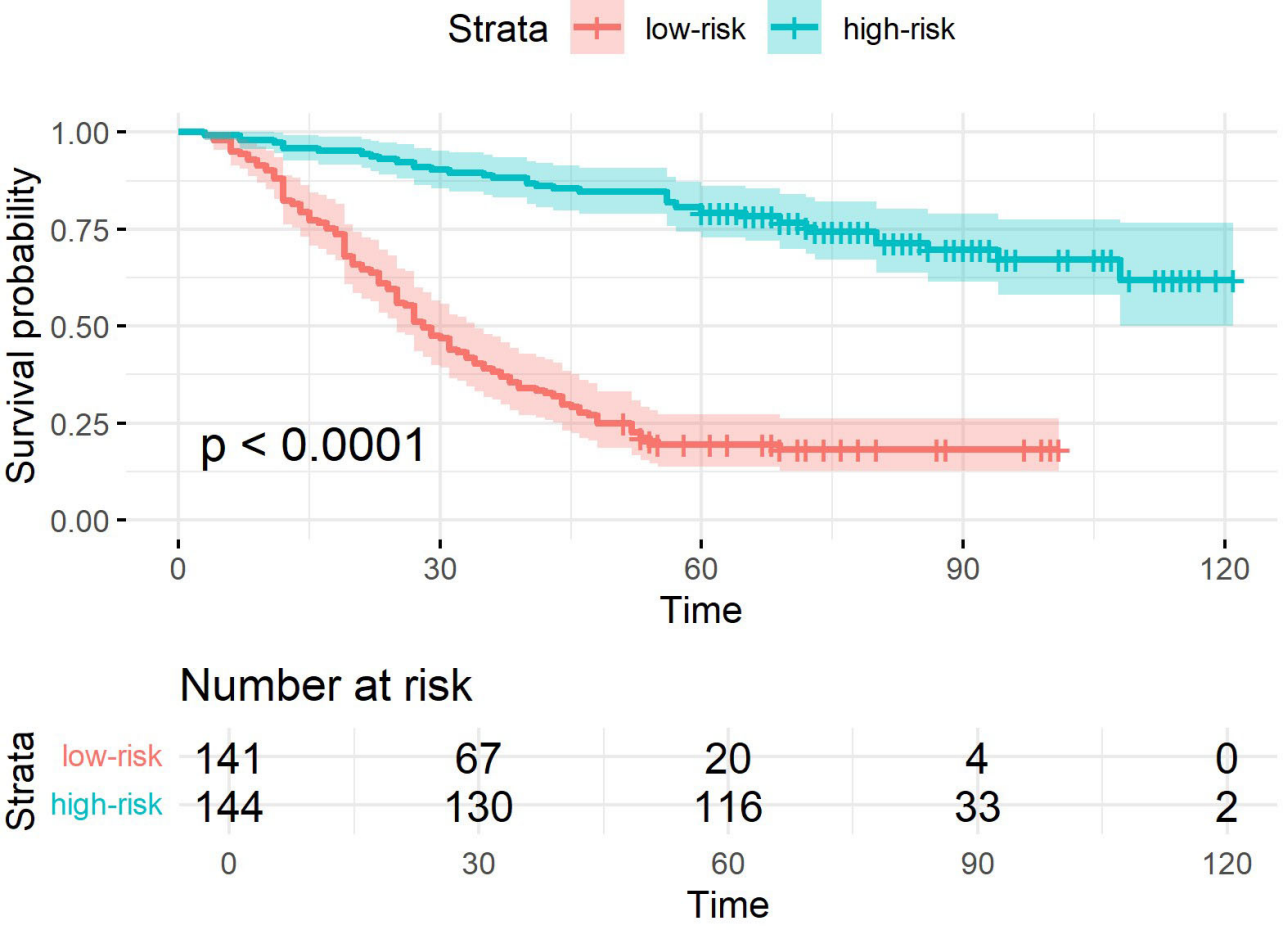
K-M curves of samples classified by ResNet-CBAM in the PJ group.

**Figure 5:**
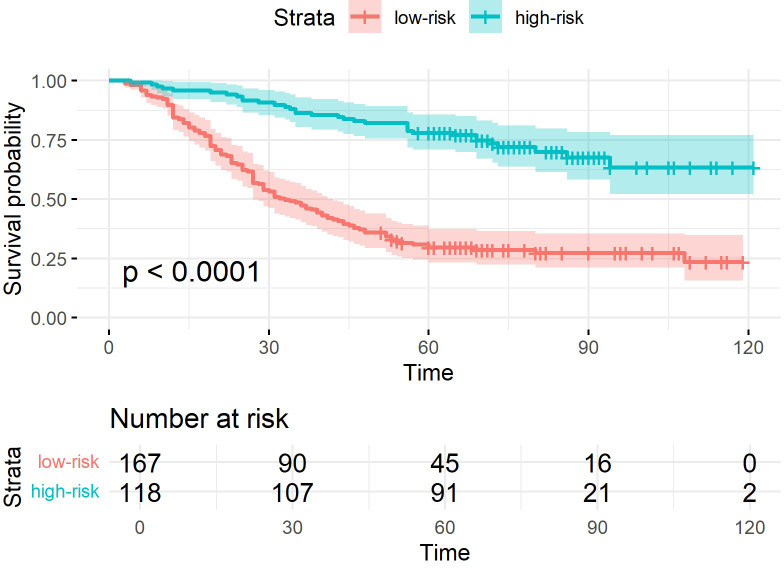
K-M curves of samples classified by ResNet-CBAM in the TM group.

**Figure 6:**
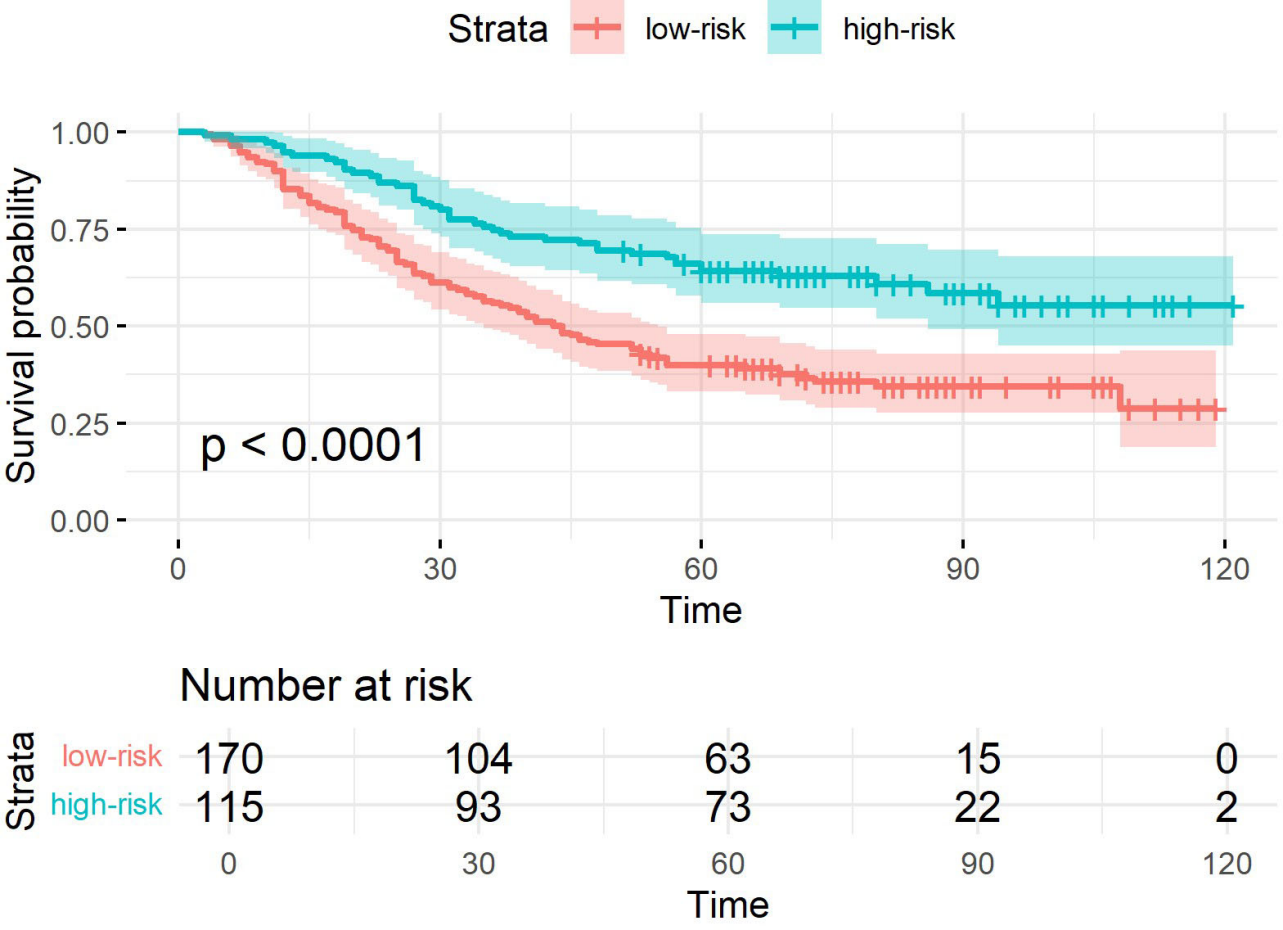
K-M curves of samples classified by ResNet-CBAM in the ZC group.

The superiority of the PJ group model was more intuitively reflected in the K-M curves (**Figures 4**–**6**). The Log-rank test demonstrated that the PJ group model effectively distinguished between high- and low-risk patients. Although the TM and ZC group models also achieved a statistically significant level of distinction, the survival risk differences between the stratified groups predicted by the PJ group model were visually more pronounced than those of the other two groups. This indicated that the PJ group model exhibited significantly stronger discriminatory power in identifying high-risk patients compared to the other models. Notably, under the PJ group model, no patients with a potential survival time exceeding 100 months (approximately 8 years) were misclassified as high-risk.

### 3.2 ResNet-CBAM model trained on PJ, TM, and ZC group datasets for predicting 1-, 3-, and 5-year survival

According to **Table 3**, the ResNet-CBAM model demonstrated significantly better training performance in the PJ group compared to the TM and ZC groups for both short- and long-term survival predictions (**Table 3**). The ZC group, intentionally designed as a control group, exhibited unsatisfactory performance even for short-term survival prediction (1-year survival; accuracy: ZC 0.810 vs. PJ 0.980, TM 0.937). This suboptimal performance was attributed to the ZC group’s training images, which retained local features of tumors and lymph nodes but deliberately removed spatial relationships and relative weights between the two. The results suggested that the inclusion of lymph node features, when misrepresented, hampered model performance and introduced noise. Moreover, as the survival prediction timeline extends, confounding factors increase, and the number of effective training samples decreases, leading to performance declines across all groups in 5-year survival predictions. Nevertheless, the PJ group, which incorporated lymph node features and tumor–lymph node spatial relationship features, achieved commendable results compared to the TM group (accuracy: PJ 0.778 vs. TM 0.719) in long-term survival prediction.

**Table 3:** Performance of ResNet-CBAM in predicting 1-, 3-, and 5-year survival in the PJ, TM, and ZC groups.

	1-Year survival			3-Year survival			5-Year survival		
	PJ	TM	ZC	PJ	TM	ZC	PJ	TM	ZC
**Accuracy**	0.981	0.937	0.810	0.833	0.755	0.691	0.778	0.719	0.667
**Precision**	0.994	0.931	0.870	0.856	0.671	0.730	0.815	0.783	0.677
**Specificity**	0.994	0.930	0.872	0.861	0.630	0.843	0.817	0.821	0.783
**Recall**	0.969	0.943	0.756	0.805	0.906	0.509	0.742	0.621	0.532
**F1-score**	0.981	0.937	0.809	0.830	0.771	0.600	0.776	0.692	0.596

**Figure 7:**
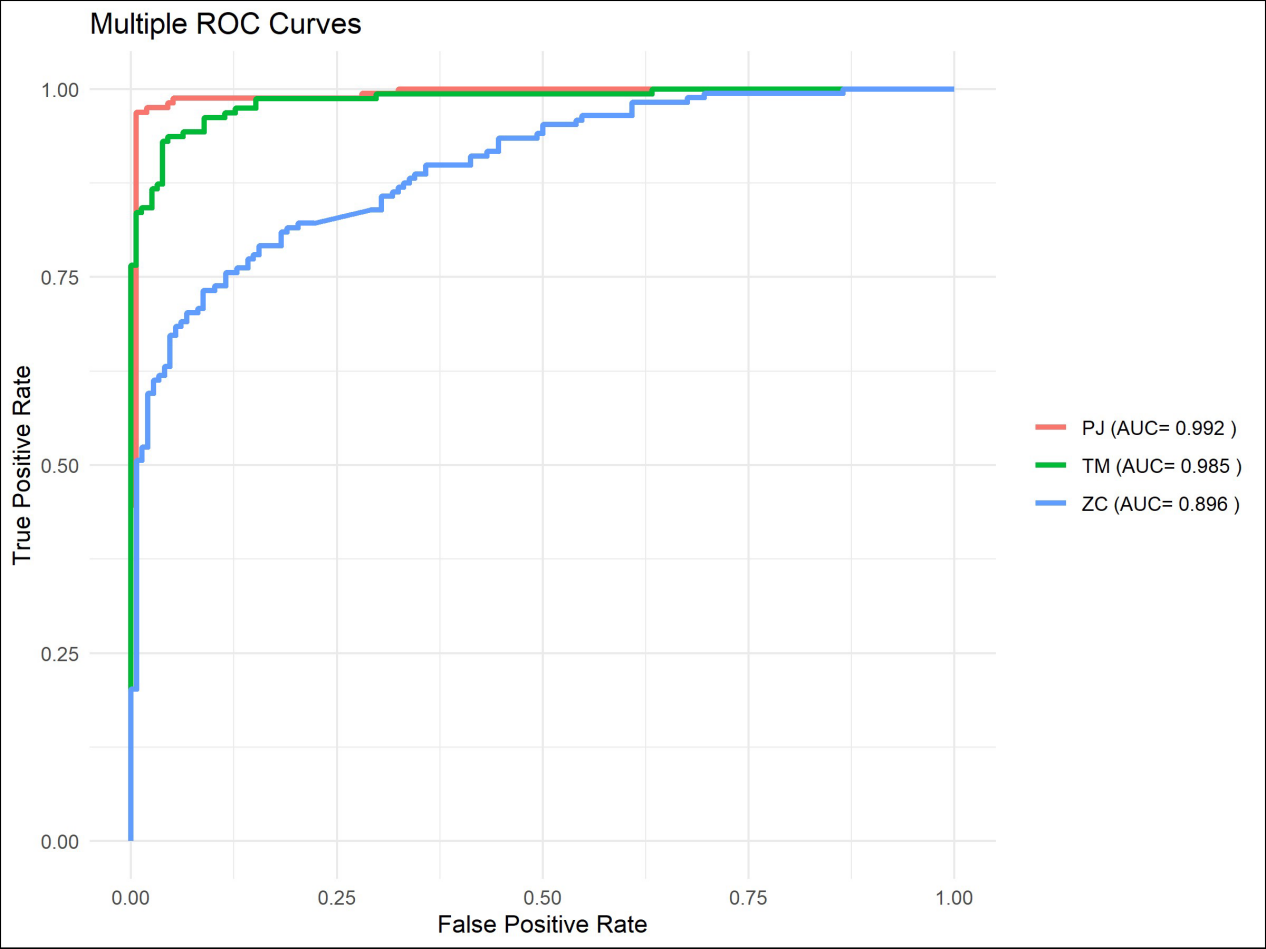
ROC curves of ResNet-CBAM in predicting 1-year survival in the PJ, TM, and ZC groups.

**Figure 8:**
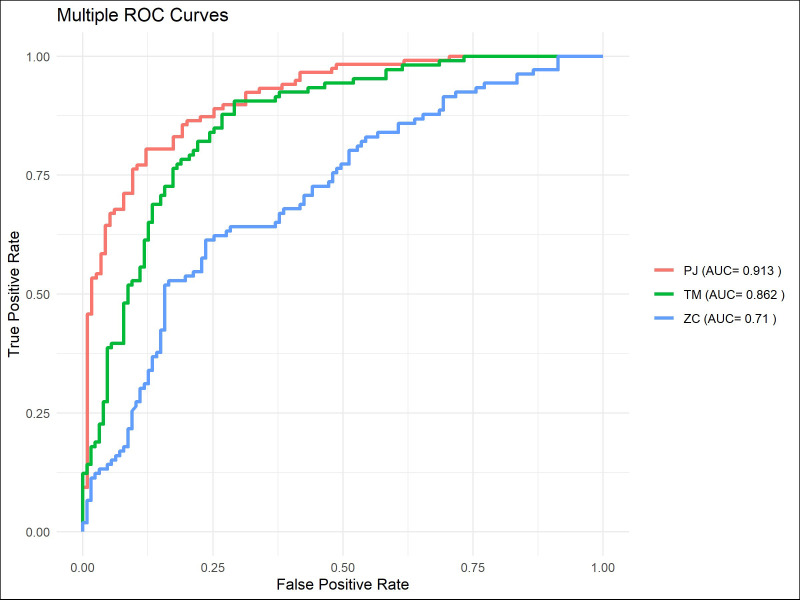
ROC curves of models in predicting 3-year survival in the PJ, TM, and ZC groups.

**Figure 9:**
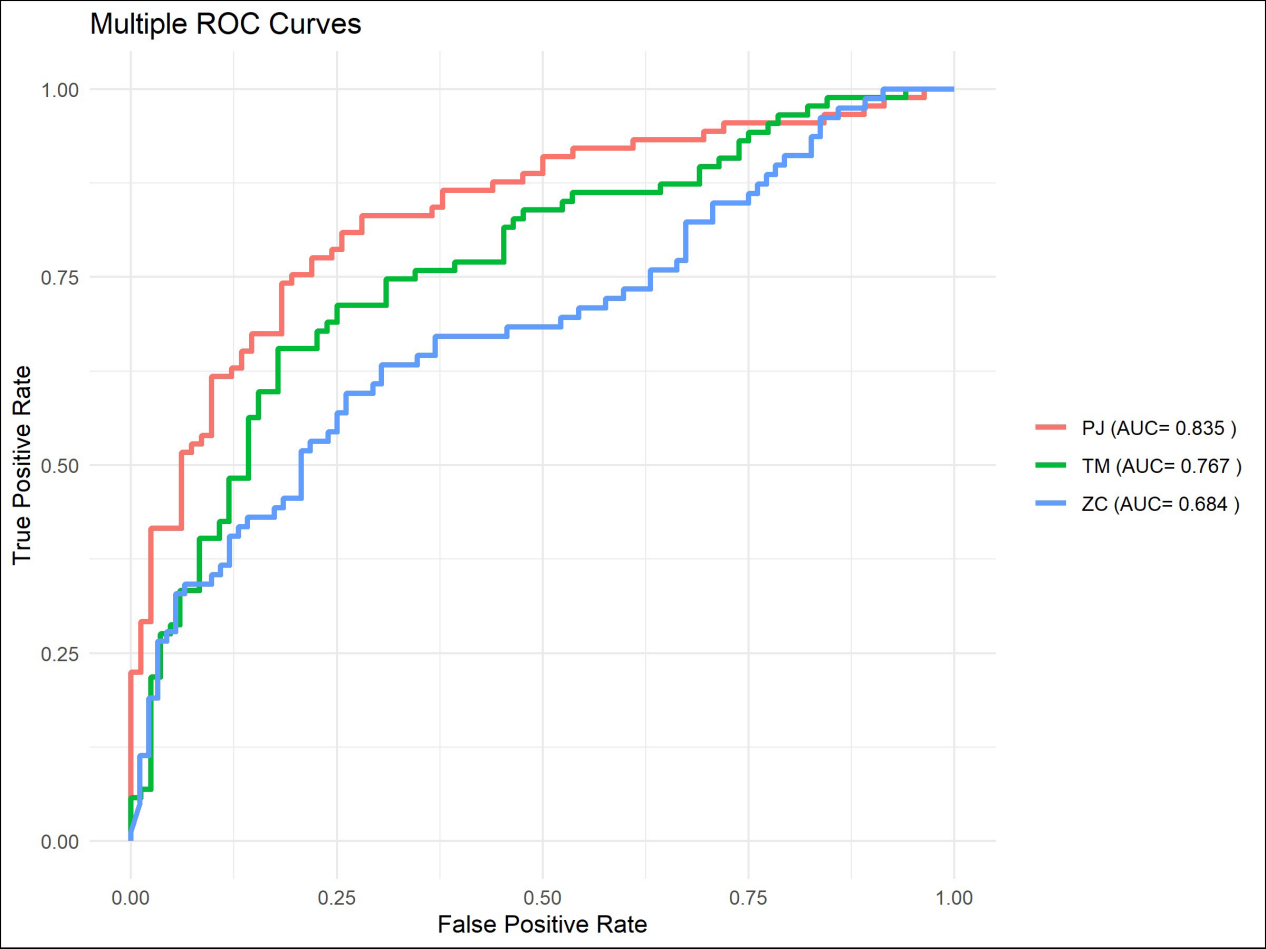
ROC curves of ResNet-CBAM in predicting 5-year survival in the PJ, TM, and ZC groups.

The ROC curve indicated that (**Figures 7**–**9**), for the 1-year short-term survival prediction task, the performance of the PJ group model was quite similar to that of the TM group (AUC: PJ 0.992 vs. TM 0.985). However, the ZC group demonstrated significantly poorer performance compared to the other two groups (AUC: ZC 0.896). The PJ group model maintained a high level of discriminative ability for long-term survival predictions (3-year AUC: PJ 0.913; 5-year AUC: PJ 0.835). In contrast, the TM and ZC group models showed a considerably weaker ability to predict long-term survival.

### 3.3 Performance of SVM, LR, and KNN for predicting 1-, 3-, and 5-year survival

**Figure 10:**
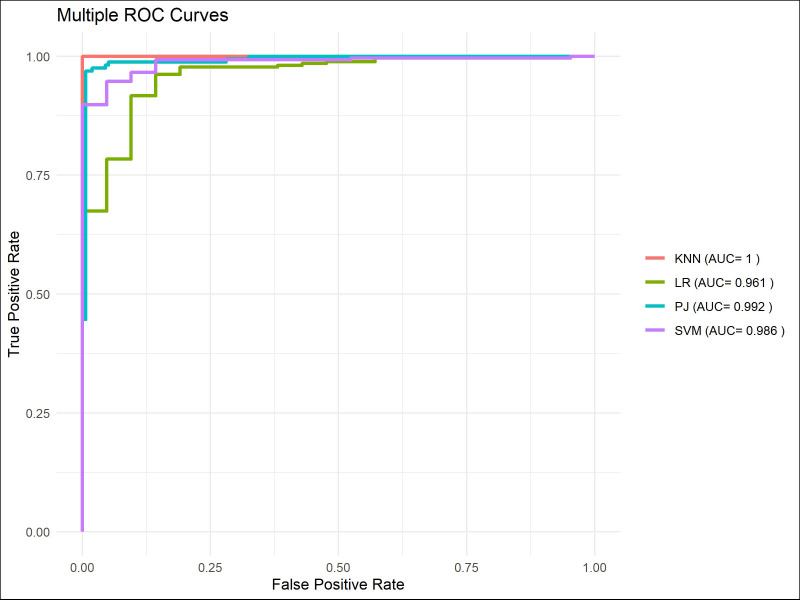
ROC curves of models for predicting 1-year survival.

**Figure 11:**
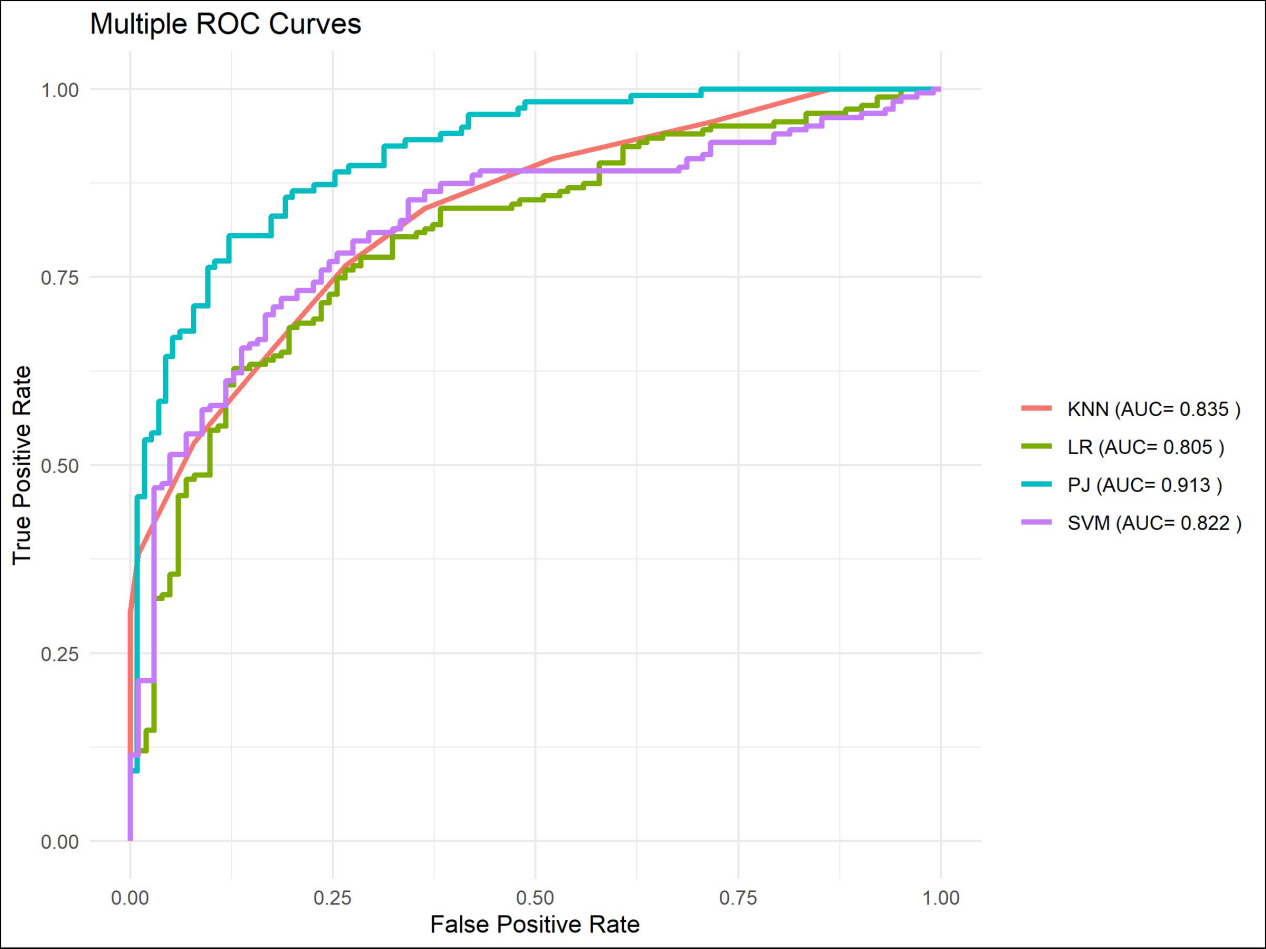
ROC curves of models for predicting 3-year survival.

**Figure 12:**
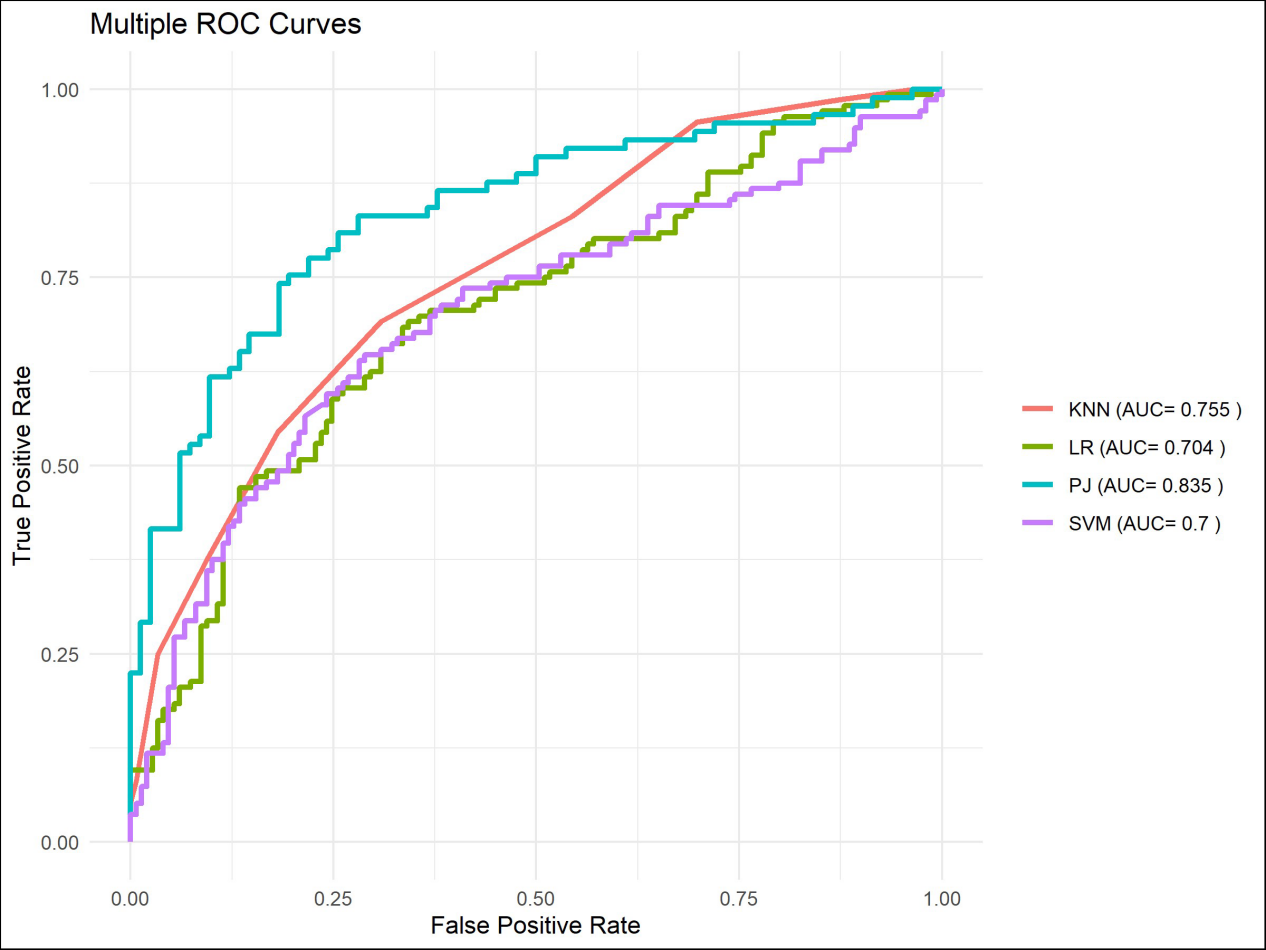
ROC curves of models for predicting 5-year survival.

The machine learning models demonstrated satisfactory performance in predicting short-term survival, with AUC scores consistently exceeding 0.95 (**Figures 10**–**12**). However, similar to the results observed in the TM group, these models were unable to maintain high discriminative power when tasked with long-term survival predictions. For instance, in the PJ group, the ResNet-CBAM achieved an AUC of 0.913 for predicting 3-year survival, while all machine learning models had AUCs below 0.9. Likewise, the ResNet-CBAM in the PJ group attained an AUC of 0.835 for 5-year survival prediction, whereas the AUCs of all machine learning models were close to or below 0.75. These findings suggest that while machine learning models can generally match the predictive accuracy of the PJ group’s ResNet-CBAM for short-term survival, they perform worse than the latter in long-term survival prediction tasks.

## 4 Discussion

With advancements in endoscopic technology, early-stage esophageal cancer cases identified through screening can now be resected without the need for additional treatment, significantly improving patient outcomes (19). However, substantial proportion of cases are still diagnosed at an advanced stage and typically require a comprehensive treatment approach combining surgery, radiotherapy, and chemotherapy (20). For these patients, the primary challenge lies not in diagnosis but in the systematic design of optimal treatment strategies and accurate prognosis evaluation. Existing clinical guidelines provide standardized measures for managing specific clinical manifestations or symptoms at different treatment stages, offering key treatment recommendations (20). Consequently, two patients with similar clinical manifestations treated at the same clinical center often receive closely aligned treatment plans and share comparable prognoses. Building on this foundation, deep learning models trained on real-world data show promise in predicting patient outcomes. By leveraging preoperative CT images and other initial diagnostic materials, these models could aid in delivering personalized prognostic predictions, potentially improving the precision of treatment planning in advanced esophageal cancer cases.

Recognizing the importance of lymph node features in CT image analysis for esophageal cancer, various studies explored ways to effectively incorporate these features into prognostic models (21, 22). Malignant lymph node enlargement was a key indicator of tumor metastasis, and lymph node ROIs provided complementary information to tumor ROIs. However, past approaches neglected the spatial relations between the two (23). The spatial distance between esophageal cancer tumors and lymph nodes was an important factor in evaluating the malignancy of the tumor. Enlarged or abnormally dense lymph nodes near the tumor often indicated that the tumor might have undergone local lymphatic spread, while distant lymph node metastasis potentially suggested a more advanced stage of the disease. The unique longitudinal lymphatic drainage structure of the esophagus meant that tumors not only could spread to nearby lymph nodes but also could extend longitudinally to mediastinal, cervical, and abdominal lymph nodes. This complex spatial relationship determined its distinct patterns of invasion and metastasis. When the positional relationship between the tumor and lymph nodes indicated extensive or multiple metastases, the prognosis was generally poor. Additionally, significant lymph node enlargement or the formation of clustered lymph node masses often suggested strong tumor invasiveness and a high tumor burden. Imaging-based textural features were also highly correlated with tumor behavior. More irregular tumor textures and subtle microlesions along invasion paths (e.g., subclinical micrometastases in lymph nodes) were associated with worse prognoses (24).

To preserve spatial relationship features across ROIs from different planes, we adopted a geometric projection method that mapped ROIs onto a shared coordinate system. This approach enabled the retention of spatial positional relationships within a unified framework. Specifically, a tumor–lymph projection plane was constructed to extract the spatial relationship features between tumors and lymph nodes. The key step involved projecting the ROIs of potentially metastatic lymph nodes onto the cross-sectional images where the tumor ROI reached its maximum size. This method effectively preserved most of the spatial relationships between tumor nodules and lymph nodes while increasing the proportion of relevant ROI areas in the training images. The PJ group demonstrated superior performance in predicting long-term survival prognosis (5-year survival) compared to other groups, such as the TM group. The accuracy of the PJ group exceeded 0.77, whereas the accuracy of the TM group was approximately 0.7. Furthermore, the PJ group outperformed in other key metrics, achieving a precision of 0.815, a specificity of 0.817, a recall of 0.742, and an F1-score of 0.776. Even in challenging long-term prognostic prediction tasks, the model trained on the PJ group dataset effectively extracted critical features and aligned with the prediction target. The superiority of the PJ group model was more intuitively reflected in the K-M curves. The survival risk differences between the stratified groups predicted by the PJ group model were visually more pronounced than those of the other two groups. This indicated that the PJ-group model exhibited significantly stronger discriminatory power in identifying high-risk patients compared to the other models.

In our study, the ZC group served as a control group with notable design flaws. Spatial information, such as the relative positions and directions of tumor and lymph node ROIs, was eliminated. Additionally, lymph node ROIs were artificially enlarged to match the size of tumor ROIs, disrupting the natural size-weighting ratio between the two. These modifications not only diminished the potential of spatial features but also overemphasized lymph node characteristics, leading to interference in the model’s training. Performance metrics clearly illustrate the limitations of the ZC group. For 3-year survival prediction, the accuracy of the ZC group was only 0.691, compared to the PJ-group and TM group, which achieved accuracies of 0.833 and 0.755, respectively. Similarly, the AUC for the ZC group was just 0.710, significantly lower than that of the other groups. As prediction periods lengthen and task complexity increases, the ZC group model becomes less effective. Overemphasis on lymph node features, combined with the elimination of spatial relationships, impeded the model’s ability to improve its prognostic performance.

Esophageal cancer is a highly malignant tumor characterized by early lymph node metastasis. The number and extent of lymph node metastases, along with the shape and volume of metastatic lymph nodes, directly impact the prognosis (25, 26). However, whether traditional CT, contrast-enhanced CT, or PET-CT is used, all these imaging methods rely on the principle of computed tomography. The extensive lymphatic drainage of the esophagus results in lymph nodes being distributed across different imaging slices. This presents significant challenges for thoracic surgeons and radiologists in image interpretation and comprehensive evaluation. Notably, the spatial relationship between lymph nodes and the tumor offers valuable insights into the tumor’s behavioral characteristics and malignancy level. Thoracic surgeons only need to outline the enlarged lymph nodes and tumor regions on different imaging slices. With the assistance of the model, they can evaluate tumor malignancy and survival risk. In this study, lymph node ROIs from different slices were projected onto the slice containing the tumor ROI within a specific CT image series. This projection method captured key tumor features, secondary lymph node features, and their spatial relationships. The results highlighted the importance of these spatial relationships in predicting the prognosis of esophageal cancer. Additionally, feature scores of tumors and lymph nodes extracted using the ResNet model were learnable by machine learning models, highlighting their potential relevance to survival prognosis (27). However, machine learning models exhibited unsatisfactory performance in long-term prognosis prediction due to the significant loss of original image information in the feature scores. In contrast, ResNet-CBAM effectively extracted hidden spatial features and fundamental local features from the tumor–lymph node projection plane. By leveraging convolutional networks and fully connected neural networks, it successfully mapped these features to survival prediction targets. The ResNet-CBAM model trained on the projection plane demonstrated robust performance in predicting both short- and long-term survival. Future plans included conducting multicenter clinical experiments to evaluate the efficacy and robustness of ResNet-CBAM, based on the tumor–lymph node projection method, across diverse patient populations. With this model, such assessments could even be integrated into preoperative examinations, enabling physicians to personalize treatment plans and closely monitor patients at higher survival risk.

## 5 Conclusion

The ResNet-CBAM model, trained on tumor–lymph projection planes, accurately distinguished esophageal cancer patients at high or low risk of death and predicted both of long- and short-term survival with superior performance compared to traditional models. By extracting features of spatial relations between the tumor and lymph nodes, it provided insights into tumor invasiveness, metastatic pathways, and other behavioral characteristics.

## Data Availability

The raw data supporting the conclusions of this article will be made available by the authors, without undue reservation.
